# Variation in Public Trust, Perceived Societal Fairness, and Well-Being before and after COVID-19 Onset—Evidence from the China Family Panel Studies

**DOI:** 10.3390/ijerph191912365

**Published:** 2022-09-28

**Authors:** Chunli Wei, Qingqing Li, Ziyi Lian, Yijun Luo, Shiqing Song, Hong Chen

**Affiliations:** 1 Center for Studies of Education and Psychology of Ethnic Minorities in Southwest China, Southwest University, Chongqing 400715, China; 2 Psychological Development Guidance Center, Guangxi University, Nanning 530004, China; 3 School of Psychology, Central China Normal University, Wuhan 430079, China; 4 Faculty of Psychology, Southwest University, Chongqing 400715, China; 5 School of Psychology, Shaanxi Normal University, Xi’an 710062, China; 6 Key Laboratory of Cognition and Personality of Ministry of Education, Southwest University, Chongqing 400715, China; 7 Chongqing Key Research Bases in Humanities and Social Sciences, Chongqing 400715, China

**Keywords:** COVID-19, public trust, perceived societal fairness, mental health

## Abstract

The sudden onset of the COVID-19 pandemic had a significant impact on all aspects of people’s lives, including their attitudes toward society and psychological well-being. This study aimed to analyze the variation in public trust, perceived societal fairness, and well-being before and after the outbreak of the coronavirus disease 2019 (COVID-19). This study used two-wave longitudinal data of 15,487 residents (2018, T1; 2020, T2) derived from the Chinese Family Panel Studies (CFPS). A repeated measures analysis of variance showed that (a) public trust, perceived societal fairness, and subjective well-being significantly improved and (b) depression significantly increased. Linear regression analysis showed that education and socioeconomic status had a significant predictive effect on public trust, perceived societal fairness, and depression; socioeconomic status had a significant predictive effect on subjective well-being. This study provides evidence and direction for current social governance, namely, policy implementation and pandemic response.

## 1. Introduction

The sudden onset of the COVID-19 pandemic has had a major impact on economic activities and social functioning. Several pandemic prevention and control measures have been implemented such as halting certain work operations, enforcing home quarantine, as well as travel restrictions. However, these measures have had a significant impact on all aspects of people’s lives, including their attitudes toward society and psychological well-being [1,2,3]. Public trust and perceived societal fairness form the foundation of attitudes toward society and play a pivotal role in mental health, human survival, and development [4,5]. Depression and subjective well-being are a direct reflection of an individual’s mental health, especially during the COVID-19 crisis. Therefore, public trust, perceived societal fairness, depression, and subjective well-being have been described to function as a social “barometer”, which provides tools and indicators for analyzing and measuring the trend of social development, contributing significantly to policy implementation, exploring social operation mechanisms, grasping social thought dynamics, and promoting long-term social governance [6].

To assess the variation in public trust, perceived societal fairness, and well-being before and after the COVID-19 onset, this study used two-wave longitudinal data of 15,487 residents (2018, T1; 2020, T2) derived from the Chinese Family Panel Studies (CFPS). Comparing the responses of those who completed the CFPS from June 2018 to March 2019, (prior to the pandemic) to those who completed the CFPS from July 2020 to December 2020 offered the setting for a natural experiment to compare how participants’ perceptions of the country, the government, and their lives changed after COVID-19 began. Most relevant studies conducted in China have been cross-sectional, and thus do not highlight the changes in public trust, perceived societal fairness, and well-being [3,7,8,9,10]. This study aimed to explore the changes in public trust, perceived societal fairness, and well-being, and verify the predictive role of education and socioeconomic status during the COVID-19 crisis. Furthermore, this study provides a basis and direction for current social governance, such as the implementation of policies and laws, as well as pandemic response.

### 1.1. Public Trust and Perceived Societal Fairness

Trust is a psychological state of willingness to take risks based on positive expectations of external intentions or behaviors [11]. The COVID-19 pandemic has highlighted the vital role that trust plays in producing and maintaining compliance with public health instructions during times of crisis [12]. It has emphasized the several varieties of trust that influence behavior when public health is at risk. People are expected to trust public health professionals to identify hazards to the public’s health with accuracy and to suggest appropriate mitigation measures. They are expected to trust officials to faithfully and rapidly implement those policies. Specifically, citizens and communities need to trust and adhere to advice from government officials and doctors. Recent studies indicate that trust in government and public health guidelines have a close relationship [13,14,15,16]. Local government officials may communicate information to ensure that citizens are aware of new public health regulations [12]. Furthermore, during the pandemic, because the effort to increase public awareness has the effect of reducing negative emotions and engaging in rescue activities, interpersonal trust is an invisible force that reduces social dangers [17]. In order to deal with COVID-19, many people are required to work with strangers. These acts come at a cost to the individual (e.g., social isolation), but are beneficial to the group as a whole (e.g., safeguarding vulnerable groups; i.e., a societal conundrum) [18]. A recent study revealed that educating individuals about the dangers the pandemic poses to their health significantly increased their trust in strangers [19].

The pandemic can influence individual trust in the government, doctors, and strangers [20]. A study found that trust in the government among Chinese residents was at an overall high level during the COVID-19 pandemic [21], as individuals were required to trust and comply with the recommendations provided by government officials and doctors to mitigate the pandemic. Trust in institutions may be enhanced instinctively among individuals in the context of a common external threat owing to limitations imposed on choices [22]. In accordance with the source model of group threat, perceptions of intergroup threat will strengthen (in)group identification processes and relations because the perceived source of a threat is crucial in forecasting its repercussions [23]. According to the source model of group threat, groups such as nations confronting external threats react by strengthening their intragroup connections [23]. Particularly, the emotional connections of individuals are strengthened through both location and national identity, developing a strong sense of community [23,24]. Thus, public trust increases after natural disasters, possibly as a result of the need for everyone to work collaboratively to overcome the tragedy [25]. Based on research conducted in the United States during the H1N1 epidemic, individuals exhibited substantial trust in public health officials [26]. Furthermore, longitudinal data analyzed in Switzerland showed that individuals initially had high levels of trust in government and public health officials. However, that trust gradually declined as the H1N1 pandemic progressed [27].

In the present study, several indices of public trust relevant to the COVID-19 pandemic were included: trust in local government officials, doctors, and strangers [12,18,19]. Local government officials and doctors are cooperating to initiate response and communicate the corresponding reasons to the public [12]. Trust and cooperation among strangers may be the key to understanding how societies are dealing with the COVID-19 pandemic [18]. These indicators cannot cover all aspects of public trust, but are representative, and the content and orientations involved are the focal issues deeply felt by the general public in a sudden pandemic [6]. Most research demonstrates that being exposed to natural disasters like earthquakes, typhoons, and volcanic eruptions strengthens relationships of trust and collaboration [25,28,29,30,31]. We, therefore, assumed that trust in local government officials, doctors, and strangers would increase after the outbreak.

Perceived societal fairness was also considered. Perceived societal fairness is the ability of an individual to perceive and judge the extent of social fairness and includes three types, namely, distributive, procedural, and interactional fairness [32,33]. An imbalance in perceived societal fairness can lead to the accentuation of social conflicts and contradictions, affecting social harmony and stability [34,35,36]. Maintaining societal cohesion by dealing with injustices would be critical in improving well-being post-pandemic [37]. The Chinese government adopted three measures, including increasing the construction of the public health system, improving the capacity of large-scale nucleic acid testing, and fully covering the cost of treating patients with coronary pneumonia, in order to maintain social harmony and stability [38]. Therefore, it was assumed that residents would experience more perceived societal fairness. Coordinated efforts among individuals, communities, and governments to combat a pandemic indicate a strong signal of cooperation, facilitating the reorganization of external and internal groups into communities of purpose. Moreover, the renewed social identity can help to coordinate and manage responses to threats while promoting adherence to commitments and norms among group members, thus facilitating national leadership behaviors [20]. An individual sense of common fate, which involves identification and positive feelings about the nation, may have also increased with the COVID-19 outbreak [23,24]. Similarly, patriotism and identification with fellow citizens increased among Americans following the 9/11 incident [39]. Thus, social identity during the COVID-19 pandemic can enhance group relations and enhance perceived societal fairness [37].

### 1.2. Depression and Subjective Well-Being

People’s mental health is likely to be impacted by the lived experience of witnessing the pandemic unfold, in addition to the social isolation and financial uncertainty brought on by the pandemic [22]. Relevant studies demonstrate that experiencing widespread calamities (e.g., natural disasters and war) instantly puts people’s mental health at risk [40,41]. First, anxiety, panic, depression, and other negative emotions may be exacerbated among individuals during the pandemic, as well as profound changes to their mental health [42,43]. Furthermore, studies found that people experienced relatively high levels of anxiety and depression after the outbreak [7,8], which is consistent with the findings underscoring high levels of fear and anxiety following the global SARS outbreak in 2003 [44,45]. Second, pandemics are considered a social threat as they reduce offline interpersonal and social contact, which undermines the subjective well-being of individuals [46], causing a specific decline in life satisfaction, future confidence, and interpersonal relationships. However, social threats also provide opportunities to enhance social cohesion and bonding [40,47], which can mitigate the negative effects of the pandemic.

To assess pre- and post-pandemic mental health, we took depression and subjective well-being as the mental health index, which reflect people’s psychological state from different aspects [48]. Subjective well-being refers to the overall judgment of an individual’s life state based on personal criteria. Furthermore, it provides a subjective, holistic, and relatively stable perspective, serving as a comprehensive psychological indicator outlining the quality of an individual’s life [49,50]. Moreover, subjective well-being emphasizes a comprehensive subjective evaluation that includes pleasurable experiences of the individual’s body and mind, emotional feelings, and satisfaction. Furthermore, two indicators of subjective well-being were examined [22,51,52]: satisfaction with life and personal well-being (future security, personal relationships, and impact of happy event). Diener et al. [51] proposed that subjective well-being includes an indicator of life satisfaction. Moreover, Cummins et al. [52] proposed a Theory of Subjective Wellbeing Homeostasis, which proposes that subjective well-being includes indicators of personal well-being, involving satisfaction with standard of living, future security, personal relationships, impact of happy/sad event, and health. Therefore, this study used life satisfaction, future confidence, interpersonal relationships, and experience of happiness to measure subjective well-being. The outbreak of the COVID-19 pandemic is likely to lead to changes in the mental health of residents. Therefore, we assumed that increased depression would be associated with decreased subjective well-being after the outbreak of COVID-19.

### 1.3. Education and Socioeconomic Status

Education is associated with public trust, perceived societal fairness, and depression. A recent study found that a higher level of education is associated with higher government trust during COVID-19 [53]. Furthermore, education has a profound impact on shaping the public’s perceived societal fairness [54]. A follow-up study found that education had a protective effect against an increase in depressive symptoms [55]. Socioeconomic status is a social classification that reflects an individual’s position in the social hierarchy, which comprises subjectively perceived social status and objective material resources measured by income, education level, and occupational status [56,57]. Socioeconomic status has an impact on all parts of an individual’s life [58], including public trust, perceived societal fairness, and well-being. The positive predictive effect of socioeconomic status on interpersonal trust has been confirmed by many cross-cultural studies [59]. Moreover, socioeconomic status affects individuals’ perceived societal fairness [60]. Some research indicated that individuals with a higher socio-economic status had greater advantages in personal health [61,62]. Evidence-based studies have shown that those with a lower socioeconomic status experienced higher rates of depressive symptoms [63], and had lower satisfaction with life [64]. In response to the COVID-19 crisis, we envision to verify the predictive role of education and socioeconomic status based on these studies.

## 2. Materials and Methods

### 2.1. Data and Study Population

The data were derived from the China Family Panel Studies (CFPS), which is a project financed by Peking University and the National Natural Science Foundation of China. The CFPS was first conducted in 2010 by its affiliate, the Institute of Social Science Survey, with a goal sample size of 16,000 homes. Furthermore, the CFPS is a multidisciplinary, national, large-scale social tracking survey including 25 autonomous regions [65]. This study linked a sample of 37,354 data points from Wave 4 of the survey, which was from June 2018 to March 2019 (hereafter referred to as T1 or 2018) with 28,590 data points from Wave 5 of the survey, which was from July 2020 to December 2020 (hereafter referred to as T2 or 2020), and 15,487 valid questionnaires were finally collected. The 15,487 participants were the same in both waves. Data were screened according to the following conditions: (1) complete data were available for the fourth and fifth waves on the measures of public trust, perceived societal fairness, depression, and subjective well-being; and (2) samples with missing values for the variables in this study were excluded. Listwise deletion was used in all analyses owing to low levels of missing data (5% on any variable) [19]. Consequently, a total of 15,487 valid data points were obtained, which consisted of 7801 men, 7686 women, 8158 living in urban areas, and 7329 living in rural areas. Their ages ranged from 16 to 90 years old (M = 45.49 years, SD = 15.22) in the fourth wave of the survey and from 18 to 92 years old (M = 47.48 years, SD = 15.22) in the fifth wave of the survey. Education levels ranged from illiterate and semi-literate (2739), elementary (2882), middle school (4786), high school (2632), college (1310), bachelor’s degree (1037), master’s degree (96), to doctoral degree (5), with values from 1 to 8.

### 2.2. Measures

#### 2.2.1. Public Trust and Perceived Societal Fairness

Participants were asked about their trust in local government officials, doctors, and strangers [12,18,19], which were scored between 0 and 10. The composite reliability coefficients were 0.77 at T1 and 0.76 at T2, which indicated acceptable reliability [66]. The factor analysis revealed that the inequality scale included two factors, one of which is five positive scoring questions and the other is three reverse scoring questions [67]. In this study, the five positively scored items, related to life during the pandemic, were used to measure individual perceptions of social justice, which were shown to be valid and reliable in previous studies [67,68]. Respondents indicate the extent of their agreement with the items using a five-point scale (strongly disagree, disagree, neither agree nor disagree, agree, and strongly agree). An example item is “There is equality in harmonious interpersonal relationships”. Higher scores indicate a stronger sense of social justice [68,69]. In this study, the composite reliability coefficients were 0.76 at T1 and 0.76 at T2.

#### 2.2.2. Depression and Subjective Well-Being

Measures included the eight-item Center for Epidemiologic Studies Depression Scale (CES-D) used to assess depressive symptoms. Participants were asked to rate the frequency of each symptom experienced throughout the previous week on a scale from 1 (“rarely or none of the time (<1 day)”) to 4 (“most or all of the time (5–7 days)”). Furthermore, previous studies showed that the eight-item CES-D scale was a valid and reliable screening measure of depressive symptoms [48,70]. In this study, the Cronbach’s alpha coefficients at T1 and T2 were 0.76 and 0.77, respectively; the composite reliability coefficients at T1 and T2 were 0.79 and 0.79, respectively. Participants’ subjective well-being was also assessed, which included life satisfaction, future confidence, interpersonal relationships, and experience of happiness [22,51,52]. Consequently, four items were selected: “satisfaction with your life” (scored between 1 and 5), “confidence in your future” (scored between 1 and 5), “the quality of your relationships” (scored between 0 and 10), and “how happy are you” (scored between 0 and 10). In order to better reflect the meaning of subjective well-being, we transformed 0 points into 1 point for “the quality of your relationships” and “how happy are you”, and performed a two-fold weighting for the points of “satisfaction with your life” and “confidence in your future”. The composite reliability coefficients at T1 and T2 were 0.82 and 0.83, respectively.

#### 2.2.3. Socioeconomic Status

Socioeconomic status was measured by two items “What is your personal income here?” and “What is your social status here?” [58]. Participants were asked about their current situation on a scale from 1 to 5 (1 = very low, 5 = very high). The two indicators were converted into standard scores according to Tan and Kraus [71] before being added. A higher level of socioeconomic status was reflected by higher scores. Socioeconomic status ranged from −1.96 to 1.89 (M = 0.00, SD = 0.88).

### 2.3. Data Analysis

SPSS software version 26 (IBM, Armonk, NY, USA) was used to manage and analyze the data. First, the existence of a common method bias was examined using Harman’s single-factor test. Thereafter, a one-way repeated measures ANOVA was conducted to explore the variation in public trust, perceived societal fairness, and well-being before and after the COVID-19 outbreak. Finally, linear regression analysis was used to examine the predictive effects of education and socioeconomic status on public trust, perceived societal fairness, depression, and subjective well-being.

## 3. Results

Harman’s single-factor test was used to assess the common method bias based on the self-reported data used. According to the exploratory factor analysis, the first factor accounted for 14.17%, which was 40% below the critical value [72], indicating that there was no significant common method bias in this study.

### 3.1. The One-Way Repeated Measures ANOVA of Public Trust and Perceived Societal Fairness

A one-way repeated measures ANOVA was conducted on public trust and perceived societal fairness, with gender, urban/rural, and age as covariates. The one-way repeated measures ANOVA results for public trust and perceived societal fairness are shown in Table 1. We chose η^2^_p_ as the effect size for the ANOVA, which can respond to the size of the difference. Notably, the value of η^2^_p_ is less than 0.04, which indicated a very small effect [73,74,75].

For public trust, the main effect of time was significant, F(1, 15,483) = 111.66, *p* = 0.000, η^2^_p_ = 0.007, 90% confidence interval; CI [76]: [0.005, 0.01], and T2 was significantly higher than T1. In terms of societal fairness, the main effect of time was significant, F(1, 15,483) = 4.50, *p* = 0.034, η^2^_p_ = 0.000, 90% CI: [0.000, 0.001], and T2 was significantly higher than T1.

### 3.2. The One-Way Repeated Measures ANOVA of Depression and Subjective Well-Being

A one-way repeated measures ANOVA was conducted on depression and subjective well-being, with gender, urban/rural, and age as covariates. The one-way repeated measures ANOVA results for depression and subjective well-being are shown in Table 1.

For depression, the main effect of time was significant, F(1, 15,483) = 4.27, *p* = 0.039, η^2^_p_ = 0.000, 90% CI: [0.000, 0.001], and T2 was significantly higher than T1. With regard to subjective well-being, the main effect of time was significant, F(1, 15,483) = 14.87, *p* = 0.000, η^2^_p_ = 0.001, 90% CI: [0.000, 0.002], and T2 was significantly higher than T1.

### 3.3. Linear Regression Analysis with Public Trust, Perceived Societal Fairness, Depression, and Subjective Well-Being as the Dependent Variables

Hierarchical multiple regression analysis was used to examine the predictive effects of education level and socioeconomic status on T2 public trust, with T1 public trust, gender, urban/rural, and age as control variables. We use the same approach to analyze perceived societal fairness, depression, and subjective well-being, with the dependent variable at T1, and gender, urban/rural, and age as control variables. As indicated in Table 2, results showed that education (β = 0.17, *p* < 0.001) and socioeconomic status (β = 0.91, *p* < 0.001) had a significant predictive effect on T2 public trust. As indicated in Table 3, the results showed that education (β = −0.08, *p* < 0.001) and socioeconomic status (β = 0.48, *p* < 0.001) had a significant predictive effect on T2 perceived societal fairness. As indicated in Table 4, the results showed that education (β = −0.22, *p* < 0.001) and socioeconomic status (β = −0.45, *p* < 0.001) had a significant predictive effect on T2 depression. As indicated in Table 5, the results showed that socioeconomic status (β = 1.99, *p* < 0.001) had a significant predictive effect on T2 subjective well-being.

## 4. Discussion

Globally, countries are taking action against COVID-19. The present study analyzed the variation in public trust, perceived societal fairness, and well-being before and after the outbreak of the COVID-19 and verified the predictive role of education and socioeconomic status. In addition to theoretical and scientific implications, these findings provide valuable information to governments, which must rapidly develop and alter COVID-19 management plans, as well as to the global population, who are confronted with this dilemma [22].

### 4.1. Variation in Public Trust and Perceived Societal Fairness

The present study found that public trust and perceived societal fairness significantly improved, which was supported by previous studies [21,25,26]. Firstly, consistent with group threat theory that perceptions of intergroup threat will strengthen (in)group identification processes and relations, a significant increase in public trust was observed in this study because external threats motivate people to unite and trust each other [23]. Secondly, the bold and decisive actions of the Chinese government, including the shutdown of the economy, have the potential to unite people in the fight against the pandemic. A recent study indicated that individuals’ trust is a critical factor at various stages of a complicated causal chain of pandemic response, such as diagnosis, regulation, promulgation, and enforcement [12]. Furthermore, public trust facilitates the implementation of pandemic prevention and control measures [20]. Studies have found that the implementation of official recommendations for preventive behaviors occurs most likely when trust in the government is high and negative feelings are low [77]. Several findings suggest that trust in local governments contributes to lower infection rates [78]. Similarly, a Dutch follow-up study found that pandemic lockdown measures increased residents’ trust in the government and science [79]. Perceived societal fairness is related to the residents’ social identities [37]. The COVID-19 pandemic outbreak has facilitated the international recognition and validation of China’s rapid response, efficient strategy, and successful experience in mitigating the pandemic. Moreover, China’s response to the outbreak has enhanced the pride of the nation. The Chinese government has created new norms within existing groups to address societal inequalities by prioritizing the safety and health of its people, thereby underscoring the concept of “life first, people first” [38]. Therefore, this may result in a very stable perceived societal fairness for residents.

### 4.2. Variation in Depression and Subjective Well-Being

The present study found that depression significantly increased among residents, which is supported by previous empirical studies [7,8]. Another surprising finding was that subjective well-being has also increased. Notably, both depression and subjective well-being had low effect sizes. First, this result may be related to the time period. In February 2020, the development trend of the pandemic was very alarming, with the most serious period in the Chinese mainland. During this stage, the negative impact of the pandemic was the most representative and intense. By April 2020, the Chinese government had curbed the development trend of the pandemic through a series of measures, with people in most areas gradually returning to work and school. As a result, the negative effects may have decreased. Data from the survey were collected from July to December 2020; therefore, individuals’ depression and subjective well-being during this period did not change significantly [42]. Second, public trust may have contributed to the stability in mood (i.e., level of depressive symptoms) and subjective well-being. A large survey conducted across several countries, including China, found that social trust was positively associated with life satisfaction [80]. Furthermore, our study found that people’s public trust and perceived societal fairness significantly improved, which may contribute to the stability in their mental health. Moreover, the improvement in subjective well-being may be impacted significantly by the positive effects of the increased public trust and perceived societal fairness. However, the adverse short-term effects of the pandemic on depression and subjective well-being were limited, which may partly be due to an increase in public trust and perceived societal fairness. Nevertheless, depressive symptoms increased after the pandemic, which is consistent with previous research showing that social threats have a negative effect on mental health [81,82]. Overall, the individuals showed resilience in mental health.

### 4.3. Predictive Role of Education and Socioeconomic Status

The present study verified the predictive role of education and socioeconomic status. Education and socio-economic status are important factors in determining public trust, perceived societal fairness, and well-being [83]. The subjective evaluation of the COVID-19 crisis responsiveness varies depending on the individual. To establish an opinion, one must have the ability and motivations to look for and analyze relevant information. Education is an essential source of information [53]. Socioeconomic status reflects an individual’s objective social resources and subjective perceptions of social status [56,57]. It is thus crucial to develop measures that correspond to the educational and socioeconomic status of individuals. The predictive role of education and socioeconomic status contributes to understanding the factors of public trust, perceived societal fairness, and well-being in crisis management. Furthermore, offering insightful information about how individuals respond to government action may be helpful in future instances.

### 4.4. Limitations and Future Research Directions

This study used public trust, perceived societal fairness, and well-being data of 15,487 residents from 2018 (T1) to 2020 (T2) from the CFPS. Overall, the residents in this study showed resilience, which is consistent with previous findings. For example, Zhou [84] underscored that the improvement in psychosocial resilience is the future direction for the social mindset of Chinese people. However, this study has several limitations. First, the data from the CFPS conducted in 2018 and 2020 were secondary. As a result, it is possible that the variables chosen for measurement did not accurately capture the concept’s meaning. Future research should employ specific subjective well-being and public trust research methods. Second, although we found a significant effect of public trust, perceived societal fairness, and well-being, the effect size was small. Future studies should concentrate on other variables that affect public trust and subjective well-being. The effects on public trust, perceived societal fairness, depression, and subjective well-being are ongoing as a result of economic, political, and social development, as well as the repeated adverse effects of the pandemic. Thus, future in-depth studies regarding this topic still need to be conducted.

First, the proportion of depressed people may increase with the recurrence of the pandemic imposed by the impact of various management policies on interpersonal socialization, such as pandemic prevention and control, and constraints on economic development [22]. Simultaneously, an immediate increase in public trust may decline over time. A follow-up study of the pandemic in Italy found that people’s sense of social responsibility increased. However, trust in the government remained largely unchanged over time owing to the impact on economic development, while trust in science and health participation decreased [85].

Second, there should be an increased focus on the differences in the ability of various groups to cope with the pandemic, focusing on assessing protective and risk factors for different groups to cope with the pandemic. For example, groups lacking social support and pre-existing underlying problems that pose a greater risk during a pandemic should be included in future research [82,86]. Furthermore, combined stressors such as unemployment or relationship instability can exacerbate the impact of a pandemic [22]. Longitudinal studies conducted in the United States after 9/11 found that most people were resilient, despite a significant proportion who developed post-traumatic stress disorder [86]. Thus, future studies should prioritize exploring approaches to help different groups cope with a pandemic [20].

Finally, the results of this study highlight the need for a unified response to the pandemic amidst adversity and the importance of gaining social acceptance for pandemic control initiatives [20,87]. This study shows that the strong response to the pandemic in China could contribute significantly to the increased public trust among the population. Furthermore, China has always adhered to the concept of building a community of human purpose. Moreover, China has been responsible for life safety and physical health within its population, which has implications for global public health and has gained widespread acceptance, most likely increasing trust in the government among residents [38].

## 5. Conclusions

The current study analyzed the variation in public trust, perceived societal fairness, and well-being before and after the outbreak of COVID-19 based on two-wave longitudinal data of 15,487 residents (2018, T1; 2020, T2) derived from the CFPS. The results showed that public trust, perceived societal fairness, and subjective well-being significantly improved and depression significantly increased. Linear regression analysis showed that education and socioeconomic status had a significant predictive effect on public trust, perceived societal fairness, and depression; socioeconomic status had a significant predictive effect on subjective well-being. Thus, this research provides evidence and direction for current social governance, with implications for policy implementation and effective pandemic response methods.

## Figures and Tables

**Table 1:** Repeated measures ANOVA of public trust, perceived societal fairness, depression, and subjective well-being (the main effect of time).

Variables	T1 (M ± SD)	T2 (M ± SD)	F	*p*	η^2^_p_	90% CI [Lower, Upper]
Public trust	14.03 ± 5.27	15.17 ± 5.08	111.66	0.000	0.007	[0.005, 0.01]
Perceived societal fairness	19.07 ± 2.64	19.15 ± 2.56	4.50	0.034	0.000	[0.000, 0.001]
Depression	13.46 ± 3.85	13.55 ± 4.06	4.27	0.039	0.000	[0.000, 0.001]
Subjective well-being	30.95 ± 5.63	30.98 ± 5.65	14.87	0.000	0.001	[0.000, 0.002]

**Table 2:** Public trust as the dependent variable.

Variables	First Level		Second Level	
	*β*	*t*	*β*	*t*
Constant	9.30 ***		9.47 ***	
T1 Public trust	0.45 ***	66.43	0.43 ***	61.92
Gender	0.27 ***	3.79	0.24 **	3.30
Urban/rural	0.01	0.18	0.02	0.20
Age	−0.01 ***	−5.69	−0.02 ***	−6.99
Education			0.17 ***	5.74
Socioeconomic status			0.91 ***	21.58
*R* ^2^	0.22		0.25	
Δ*R*^2^	0.22 ***		0.02 ***	
F	1118.42 ***		849.82 ***	

Note: ** *p* < 0.01, *** *p* < 0.001.

**Table 3:** Perceived societal fairness as the dependent variable.

Variables	First Level		Second Level	
	*β*	*t*	*β*	*t*
Constant	13.11 ***		14.25 ***	
T1 Perceived societal fairness	0.31 ***	42.43	0.29 ***	39.00
Gender	0.22 ***	5.59	0.25 ***	6.61
Urban/rural	−0.31 ***	−8.00	−0.17 ***	−4.16
Age	0.00	1.95	−0.01 ***	−5.29
Education			−0.08 ***	−5.29
Socioeconomic status			0.48 ***	21.42
*R* ^2^	0.12		0.14	
Δ*R*^2^	0.12 ***		0.03 ***	
F	509.15 ***		432.81 ***	

Note: *** *p* < 0.001.

**Table 4:** Depression as the dependent variable.

Variables	First Level		Second Level	
	*β*	*t*	*β*	*t*
Constant	7.26 ***		8.27 ***	
T1 Depression	0.50 ***	66.85	0.48 ***	63.66
Gender	−0.37 ***	−6.43	−0.32 ***	−5.64
Urban/rural	−0.42 ***	−7.32	−0.33 ***	−5.40
Age	−0.00	−0.32	−0.00	−1.69
Education			−0.22 ***	−9.28
Socioeconomic status			−0.45 ***	−13.37
*R* ^2^	0.24		0.25	
Δ*R*^2^	0.24 ***		0.01 ***	
F	1216.65 ***		865.99 ***	

Note: *** *p* < 0.001.

**Table 5:** Subjective well-being as the dependent variable.

Variables	First Level		Second Level	
	*β*	*t*	*β*	*t*
Constant	13.54 ***		17.25 ***	
T1 Subjective well-being	0.52 ***	76.00	0.43 ***	63.74
Gender	0.11	1.48	0.12	1.58
Urban/rural	−0.32 ***	−4.18	0.02	0.21
Age	0.03 ***	11.95	0.01 *	2.17
Education			0.02	0.61
Socioeconomic status			1.99 ***	44.71
*R* ^2^	0.28		0.37	
Δ*R*^2^	0.28 ***		0.08 ***	
F	1532.81 ***		1487.42 ***	

Note: * *p* < 0.05, *** *p* < 0.001.

## Data Availability

Original data in this study are obtained from the Institute of Social Science Survey of Peking University and are available at http://www.isss.pku.edu.cn/cfps/index.htm (registration and approval needed, accessed on 8 January 2022).
